# Left Bundle Branch Block Tachycardia: A Nightmare Confluence of Tachycardia and Bradycardia

**DOI:** 10.19102/icrm.2026.17063

**Published:** 2026-06-15

**Authors:** Sudipta Mondal, Nadeem Afroz Muslim, Tapan Kr Matia, Debargha Dhua

**Affiliations:** 1 Department of Cardiology, The Mission Hospital, Durgapur, India; 2 Department of Cardiology, Neotia Getwel Multispecialty Hospital, Siliguri, India

**Keywords:** Atrial tachycardia, infra-Hisian Wenckebach, left bundle branch block, right bundle branch block, supraventricular tachycardia

## Abstract

A frail nonagenarian presented with incessant wide complex tachycardia exhibiting a left bundle branch block (LBBB) morphology. The arrhythmia was transiently responsive to adenosine boluses but proved refractory to direct-current cardioversion, exhibiting a pattern of immediate reinitiation. Upon transient termination, the baseline electrocardiogram demonstrated sinus rhythm with varying degrees of LBBB aberrancy. Intracardiac electrogram analysis was used to narrow the differential diagnosis of the tachycardia mechanism. Crucially, post-termination recordings documented a rare phenomenon of alternating bundle conduction during bradycardia. This was characterized by a severely diseased infra-Hisian conduction system with an H–V interval of 306 ms—representing one of the longest documented intervals in the literature. This case highlights the capacity for extremely slow, albeit tenuous, conduction within a profoundly diseased conduction system.

A frail nonagenarian was admitted having presented with incessant wide complex tachycardia with a left bundle branch block (LBBB) morphology at a rate of 185 bpm **(Figure 1A)**. Baseline electrocardiography (ECG) and ambulatory ECG revealed a predominant complete LBBB pattern **(Figure 1B)** with transient QRS narrowing during periods of longer coupling **(Figure 2)**, suggestive of frequency-dependent conduction improvement. Boluses of adenosine terminated the tachycardia, which then reinitiated shortly after—a pattern that continued incessantly **(Figure 3A)**. Direct-current cardioversion attempts were unsuccessful. Following a transient termination of the tachycardia, the patient’s baseline electrocardiogram revealed a sinus rhythm with varying degrees of LBBB aberrancy **(Figures 3B and 3C)**. Given the incessant nature of the tachycardia, the patient was scheduled for an electrophysiology study. Institutional standard procedural protocol involves initial placement of the coronary sinus (CS) catheter. In this case, a routine CS cannulation was anticipated; however, the anatomy proved unexpectedly challenging. During the manipulation of the CS catheter, a presumed injury to the right bundle (RB) branch led to a prolonged asystolic period **(Figure 4A)**, necessitating intubation and supportive care. Subsequently, the tachycardia terminated spontaneously **(Figure 4B)**, and atrioventricular (AV) conduction resumed in a distinctive 2:1 RB branch block pattern with intermittent LBBB morphology **(Figure 5A)**. Intracardiac electrogram recordings revealed the possible underlying mechanisms of the arrhythmia **(Figures 5B and 5C)**. The patient underwent implantation of a permanent pacemaker and was medically managed with β-blockers and amiodarone to control the arrhythmia.

**Figure 1:**
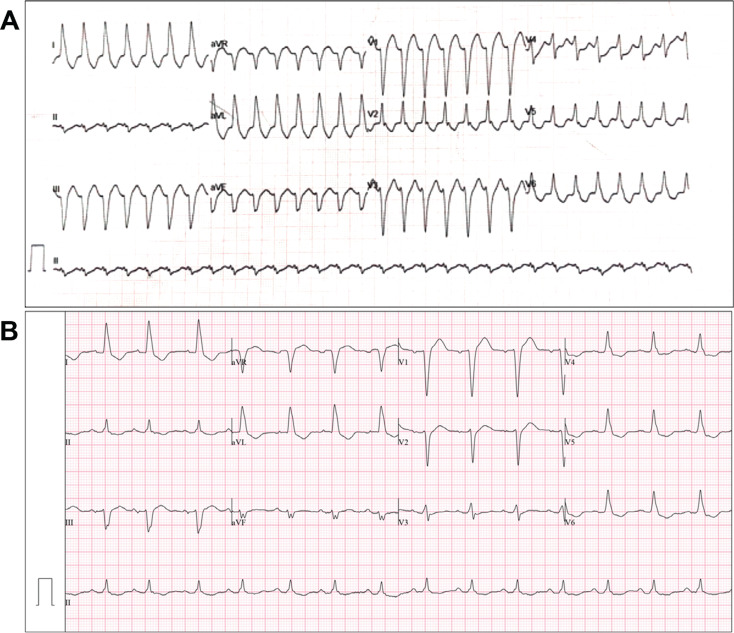
**A:** Wide complex tachycardia at 185 bpm with a left bundle branch block (LBBB) morphology. P-waves are not clearly discernible, suggesting that they may be obscured by or buried within the QRS complexes. Possible diagnoses are typical atrioventricular nodal re-entrant tachycardia (AVNRT), atypical AVNRT, atrioventricular reciprocating tachycardia (AVRT) with LBBB aberrancy, atriofascicular pathway-related antidromic AVRT (Mahaim tachycardia), atrial tachycardia with LBBB aberrancy, and rarely ventricular tachycardia (VT) with 1:1 ventriculoatrial conduction. The presence of varying degrees of rate-related LBBB aberrancy and the existing algorithms (Vereckei’s algorithm, Brugada’s algorithm, Pava’s criteria) for differentiating VT versus supraventricular tachycardia (SVT) favor (rather than clinch) the rhythm diagnosis of SVT. **B:** Baseline electrocardiogram showing complete LBBB (change in V2 is likely due to different positions of the lead taken at different times).

**Figure 2:**
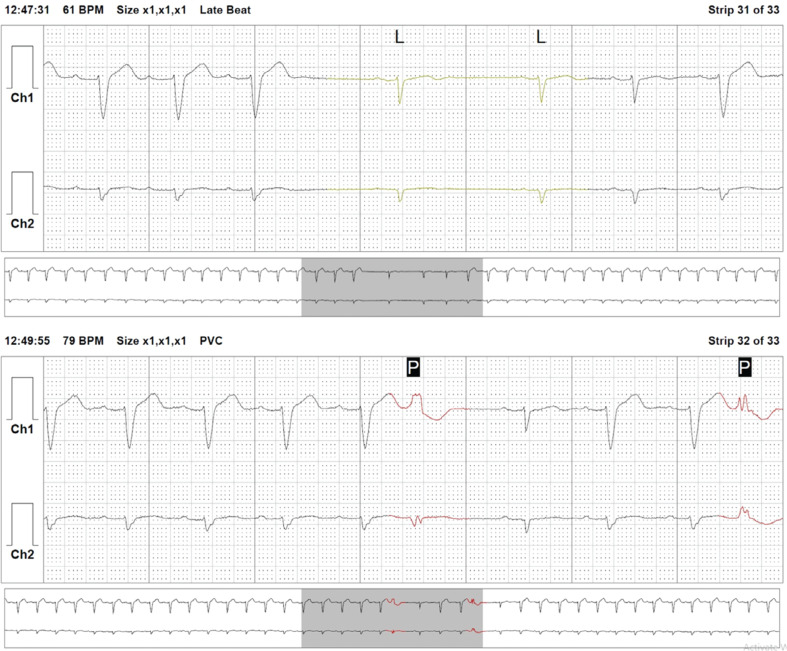
Ambulatory electrocardiogram revealed a predominant complete left bundle branch pattern with transient QRS narrowing during periods of longer coupling, suggestive of frequency-dependent conduction improvement. *Abbreviation:* PVC, premature ventricular complex.

**Figure 3:**
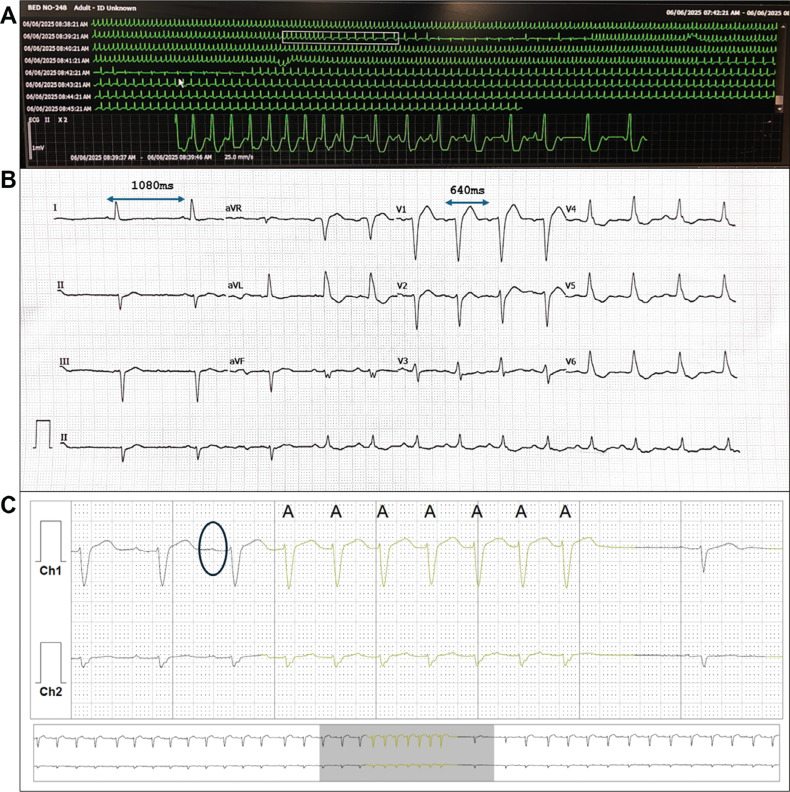
**A:** Tachycardia requiring multiple boluses of adenosine for termination, which recurs after a brief period of sinus rhythm. While this pattern is common with atrioventricular nodal re-entrant tachycardia (AVNRT) or atrioventricular reciprocating tachycardia, certain monomorphic ventricular tachycardias (VTs), particularly idiopathic VTs (adenosine-sensitive right ventricular outflow tract VT), can be transiently terminated by adenosine. While the wide complex tachycardia strongly points toward supraventricular tachycardia with aberrancy rather than monomorphic VT—supported by multiple differentiation algorithms (Vereckei’s algorithm, Brugada’s algorithm, Pava’s criteria), a baseline left bundle branch block (LBBB) that mirrors the tachycardia’s QRS morphology, and a reproducible termination following adenosine administration—this clinical picture remains highly suggestive rather than entirely definitive. **B:** Variable QRS widening observed during sinus rhythm suggests either rate-related aberrancy or intermittent pre-excitation. The initial three QRS complexes display a left anterior hemiblock morphology, indicating that the refractory period of the left anterior fascicle lies somewhere between 640 and 1080 ms. **C:** A likely atrial premature complex (indicated by a circle) triggers a tachycardia without a significant change in the P–R interval during the first cycle (from Holter monitoring). This finding raises the possibility of an AVNRT or an adenosine-sensitive atrial tachycardia with an LBBB aberrancy. The lack of change in QRS morphology between sinus rhythm and the tachycardia cycles makes Mahaim tachycardia an unlikely diagnosis.

**Figure 4:**
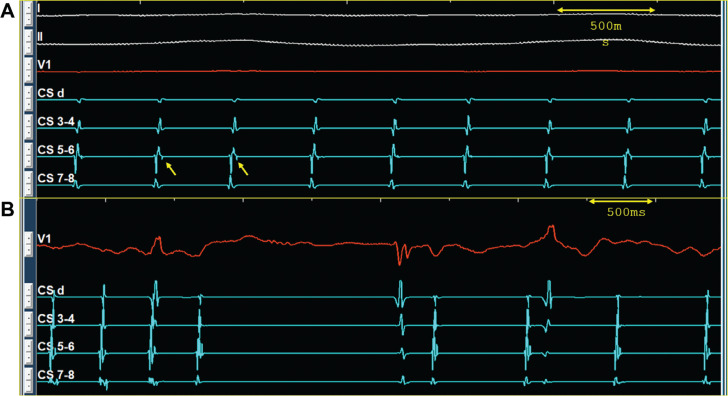
**A:** Electrophysiology study began with incessant tachycardia. Coronary sinus (CS) entry was difficult without success. Hence, the catheter was floating in the right atrium, with CS 5,6 being around Koch’s triangle anatomically. During decapolar catheter manipulation, complete heart block was observed with prolonged asystole with continuation of supraventricular tachycardia with a cycle length of 320 ms. The block was likely infra-Hisian, as noted by the presence of the His signal on CS 5,6 (arrows) with a normal A–H interval. The variable A–A interval suggests an adenosine-sensitive atrial tachycardia rather than an atrioventricular nodal re-entrant tachycardia with infra-Hisian block. Continuation of tachycardia without the involvement of the ventricle rules out atrioventricular reciprocating tachycardia. **B:** Spontaneous termination of the tachycardia.

**Figure 5:**
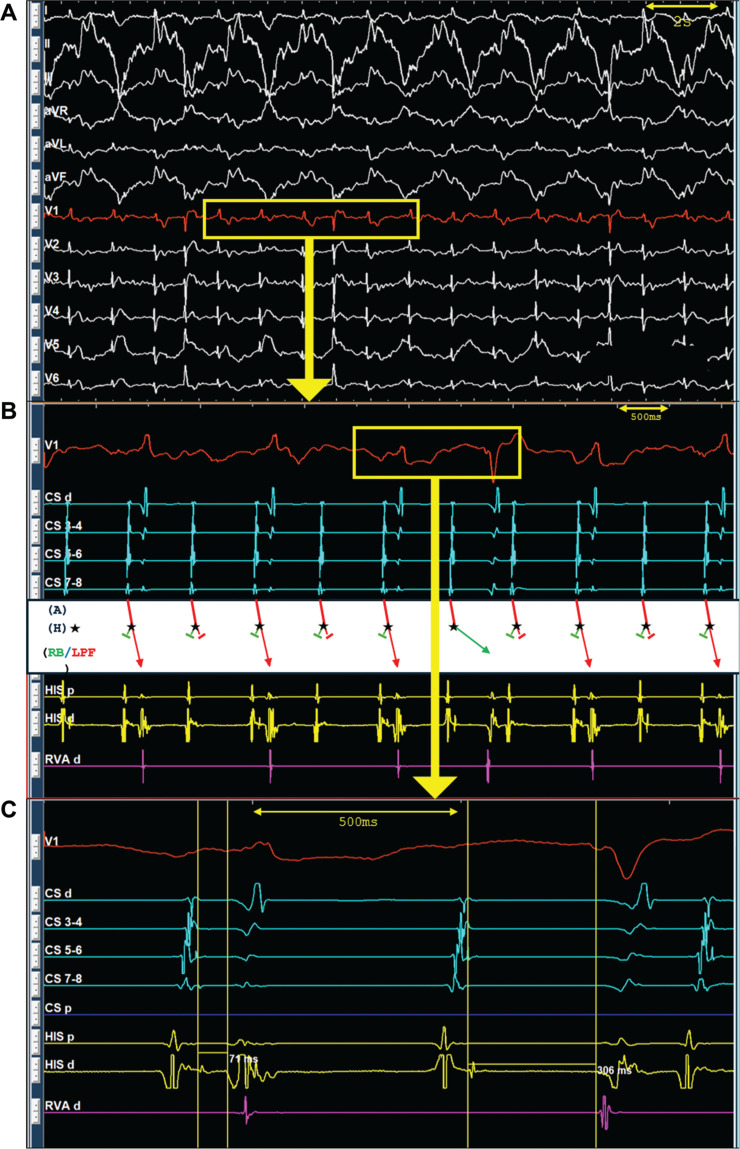
**A:** Sinus rhythm with variable atrioventricular (AV) conduction is seen following the termination of tachycardia. A 2:1 AV conduction with a right bundle (RB) branch block and left anterior deviation morphology suggests conduction through the left posterior fascicle (LPF). An intermittent left bundle branch block indicates RB conduction. **B:** The A–H interval remains fixed and normal. An infra-Hisian 2:1 LPF conduction is noted with an H–V interval of 71 ms. Intermittently, the RB conducts, showing a significantly prolonged H–V interval of 306 ms. This sequence was observed in a repetitive fashion and probably indicated an RB peeling of refractoriness (in a diseased RB). It represents an example of alternating fascicular conduction, demonstrating the possibility of conduction even with a very prolonged H–V interval. **C:** A magnified view shows the fixed A–H interval and alternating H–V intervals of 71 ms (LPF conduction in a 2:1 fashion) and 306 ms (intermittent RB conduction). The persistence of this pattern led to the implantation of a dual-chamber pacemaker. The patient remains symptom-free on a 6-month follow-up while on a β-blocker and low-dose amiodarone.

The central purpose of this case report is to document the rare phenomenon of alternating bundle conduction during bradycardia. Consent was obtained from the patient, in line with the Committee on Publication Ethics guidance for the publication of the case report. As demonstrated in **Figure 5**, the left posterior fascicle conducts on alternate beats; notably, when the RB recovers—during the beats where the left bundle is not conducting—it does so with an extremely prolonged H–V interval of 306 ms. To the best of our knowledge, this represents one of the longest documented H–V intervals in the literature, illustrating that a severely diseased infra-Hisian conduction system can maintain slow, albeit tenuous, conduction. This case did not allow us to study the definite mechanism of tachycardia for obvious reasons depicted already. The brief tachycardia study was included primarily to provide clinical context and narrow the differential diagnosis (likely atrial tachycardia or AV nodal re-entrant tachycardia) rather than to provide a definitive mechanistic study. A follow-up 12-lead electrocardiogram showed atrial-paced, ventricular-sensed rhythm with left anterior fascicular block only **(Figure 6)**. A repeat electrophysiology study is advised only should symptomatic tachycardia prove refractory to medical therapy.

**Figure 6:**
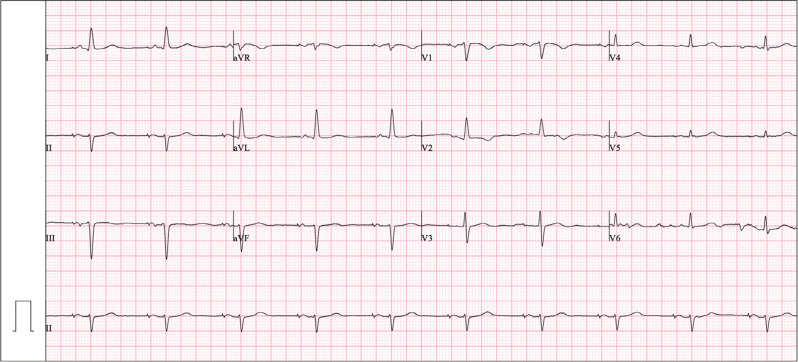
Follow-up electrocardiogram showing an atrial-paced, ventricular-sensed rhythm with left anterior fascicular block only.

